# Patients’ anxiety, fear, and panic related to coronavirus disease 2019 (COVID-19) and confidence in hospital infection control policy in outpatient departments: A survey from four Thai hospitals

**DOI:** 10.1017/ice.2020.1240

**Published:** 2020-10-07

**Authors:** Anucha Apisarnthanarak, Chanida Siripraparat, Piyaporn Apisarnthanarak, Michael Ullman, Pavarat Saengaram, Narakorn Leeprechanon, David J. Weber

**Affiliations:** 1 Division of Infectious Diseases, Faculty of Medicine, Thammasat University, Prathum Thani, Thailand; 2 Manorom Hospital, Bangkok, Thailand; 3 Division of Diagnostic Radiology, Department of Radiology, Faculty of Medicine Siriraj Hospital, Mahidol University, Bangkok, Thailand; 4 Research and Consulting Service, Michael Ullmann Consulting, Baltimore, Maryland, United States; 5 Bumrungrad Hospital, Bangkok, Thailand; 6 Department of Ophthalmology, Rutnin Hospital, Bangkok, Thailand; 7 Division of Infectious Diseases, University of North Carolina, Chapel Hill, North Carolina, United States

*To the Editor—*The emergence of the coronavirus disease 2019 (COVID-19) pandemic has disrupted day-to-day patient life with limitations to social practices (eg, physical distancing, mask wearing, and frequent hand hygiene).^1^ These limitations, together with widespread anxiety and stress, have generated a mental health crisis among patients.^2^ Anxiety, fear and panic related to COVID-19 may result in strong emotions and reactions.^1–3^ Therefore, we conducted a survey to evaluate COVID-19–associated patient emotions and confidence in hospital infection prevention (IP) and IP behaviors in outpatient departments.

This survey was performed at 2 university hospitals and 2 private hospitals from May 1 to May 30, 2020. To represent multiple patient populations, patients visiting 3 outpatient departments (general medicine, ophthalmology, and radiology) were invited to participate in the study and were interviewed using a standardized data collection tool. The first 50 patients who filled out the survey in each hospital were included in the data analysis. The data collected included patient demographics, perception of risks to contract COVID-19, confidence in policy and preparedness plan for COVID-19, sources of knowledge, and emotions evoked by COVID-19, and IP practices (eg, hand hygiene, wearing a mask, and physical distancing). Respondents rated their confidence level on knowledge and hospital preparedness plan on a scale from 1 to 5 (1, “no confidence” to 5, “very confident”) as well as changing in IP behaviors on a scale from 1 to 5 (1, “never use” to 5, “always use”). IP behavior changes (eg, hand hygiene, wearing a mask, and physical distancing) were defined as a rating of 4 (almost always) or 5 (always). We used the Generalized Anxiety Disorder 7-item (GAD-7) scale to categorize anxiety, self-rated fear, and panic on a scale from 1 to 10 (1, “no fear/panic” to 10, “extreme fear/panic”). The categorization of the GAD-7 score followed the original scale (ie, 0–4, minimal anxiety; 5–9, mild anxiety; 10–14, moderate anxiety; and >14, severe anxiety),^4^ and self-reported fear >6 was categorized as fear of COVID-19.

All analyses were performed using SPSS version 19 software (IBM, Armonk, NY). The χ^2^ or Fisher exact test was used to compare categorical variables. The Mann-Whitney U test was used for continuous data. All *P* value were 2-tailed, and *P* < .05 was considered statistically significant. Multivariate analysis was used to evaluate factors associated with emotions and impact of emotions on IP practices.

In total, 200 patients participated in this survey (n = 50 patients per hospital). The median age of respondents was 45 years (range, 15–92), and 138 of 200 participants (70%) were women. Some patients reported having had contact with COVID-19 patients or a patient under investigation (19 of 200, 9.6%). Anxiety, fear, and panic related to COVID-19 were reported by 181 of 200 (90%), 89 of 200 (45%), and 82 of 200 (41%), respectively. Feelings of discrimination and stigma against COVID-19 patients were reported by 113 of 200 (57%) and 107 of 200 (54%), respectively. Social media (164 of 200, 83%) was the most common source of COVID-19 information among patients (Table 1). There were no differences in patients’ characteristics between private and university hospitals.

**Table 1.** Patients Characteristics, Emotions, Confidence in Hospital Infection Prevention Practices at Outpatient Departments During the COVID-19 Pandemic

Variables	Total (N = 200), No. (%)
Age, median y (range)	45 (15–92)
Sex, female	138 (69)
**Occupation**	
Employee	41 (20.7)
Business man	35 (17.3)
Government worker	22 (11)
Others^a^	102 (51)
**Type of mask**	
Cloth mask	113 (57)
Surgical mask	71 (36)
N95 mask	11 (5.6)
Others^b^	5 (2.5)
Contact with COVID-19 patients or patient under investigation	19 (19.6)
Fear for contracting COVID-19	89 (45)
Panic for being contracting COVID-19	82 (41.4)
Confidence in hospital preparedness policy	175 (88)
Confidence in hospital hand hygiene policy	196 (99)
Confidence in wearing mask policy at outpatient department	187 (94)
Confidence in social distancing policy at outpatient department	163 (82)
Confidence in COVID-19 knowledge	150 (76)
**Source of COVID-19 information**	
Social media	
Line app	164 (83.5)
Facebook	135 (67)
Instragram	154 (77)
Government news	171 (86)
Television news	174 (87)
Feeling of discrimination	113 (57)
Feeling of stigmatization	107 (54)
**GAD-7 Score**	
Mild anxiety	155 (78)
Moderate anxiety	15 (7.6)
Severe anxiety	11 (5.6)
**Changing in infection control behavior**	
Hand washing	140 (70)
Wearing mask	124 (62)
Social distancing at workplace and outpatient department	159 (79)

Note. PPE, personal protective equipment; HCP, healthcare personnel; GAD-7, Generalized Anxiety Disorder 7-items.

a Students, healthcare personnel, housewife, unemployment, self-employed.

b Self-made mask.

Most patients (175 of 200, 88%) expressed confidence in the overall hospital IP policy. Patient confidence in policies was as follows: hand hygiene (196 of 200, 99%), physical distancing (163 of 200, 82%), and mask wearing in the outpatient department (187 of 200, 94%). Only 159 of 200 (80%) reported that the hospital had adequate PPE for patients. Most patients reported changing behavior with more frequent hand hygiene (140 of 200, 70%), wearing mask at workplace or hospital (124 of 200, 62%), complying with physical distancing at workplace or hospital (159 of 200, 79%), and 150 of 200 (76%) expressed confidence in their knowledge of severe acute respiratory coronavirus virus 2 (SARS-CoV-2) transmission (Table 1).

By multivariate analysis, no factor was associated with anxiety, fear, and panic. However, patients who reported anxiety and panic were more likely to wear a mask at the workplace or hospital (adjusted odds ratio [aOR], 5.4; 95% CI, 1.7–45.5), and patients who reported fear were more likely to wear mask at the workplace or hospital (aOR, 6.4; 95% CI, 1.8–52.6) and to wash hands more frequently (aOR, 5.7; 95% CI, 1.7–51.5). Notably, patients who reported having good information regarding SARS-CoV-2 transmission were more likely to comply with physical distancing policy at the workplace or hospital (aOR, 4.2; 95% CI, 1.2–15.4), to wash hands more frequently (aOR, 5.9; 95% CI, 1.5–22), and to wear a mask at the workplace or hospital (aOR, 4.9; 95% CI, 1.3–18.9).

Our findings suggest that most patients were overwhelmed with anxiety, fear, and panic during the COVID-19 epidemic, despite a high level of confidence in hospital IP practices. Although these emotions as well as information regarding SARS-CoV-2 transmission led to changing their behavior (eg, hand hygiene, wearing a mask and physical distancing), we found that a significant proportion of patients were feeling discrimination and stigma toward COVID-19 patients. Thus, education on SARS-CoV-2 transmission should be provided in a way that does not trigger feelings of fear, anxiety, panic, discrimination, and stigmatization because these feelings may lead to violence in the community toward COVID-19 patients.^5^

Most patients had confidence in the hospital preparedness policy in outpatient departments; however, the level of changes in IP practices was still less than ideal. Therefore, additional strategies to enhance the level of IP practices in outpatient department are needed. Furthermore, several mask types are used in these patient populations (eg, surgical masks and N95 respirators). Education to emphasize the use of nonmedical masks among patients during outpatient visits is necessary.

Despite the limitation of self-reported survey and the sample size in this study, our study supports the need for hospitals to continuously provide information regarding SARS-CoV-2 transmission and an adequate supply of masks as well as emphasizing education on IP practices in outpatient departments. Additional studies on the impact of SARS-CoV-2 transmission knowledge on appropriate IP behaviors as well as perception of discrimination and stigmatization against COVID-19 patients should be conducted.
